# *Pseudomonas aeruginosa’s* greenish-blue pigment pyocyanin: its production and biological activities

**DOI:** 10.1186/s12934-023-02122-1

**Published:** 2023-06-08

**Authors:** Ahmed A. Abdelaziz, Amal M. Abo Kamer, Khaled B. Al-Monofy, Lamiaa A. Al-Madboly

**Affiliations:** Department of Pharmaceutical Microbiology, Faculty of Pharmacy, Tanta University, Tanta, Egypt

**Keywords:** Bioprospecting, *Pseudomonas aeruginosa*, Bacterial pigments, Pyocyanin

## Abstract

A subject of great interest is the bioprospecting of microorganisms and their bioactive byproducts, such as pigments. Microbial pigments have various benefits, including being safe to use due to their natural makeup, having therapeutic effects, and being produced all year round, regardless of the weather or location. *Pseudomonas aeruginosa* produces phenazine pigments that are crucial for interactions between *Pseudomonas* species and other living things. Pyocyanin pigment, which is synthesized by 90–95% of *P. aeruginosa*, has potent antibacterial, antioxidant, and anticancer properties. Herein, we will concentrate on the production and extraction of pyocyanin pigment and its biological use in different areas of biotechnology, engineering, and biology.

## Background

Microorganisms yield secondary metabolites, including bacterial pigments, to support their protection and persistence [1]. Many bacteria produce pigments that exhibit several functions, including defending against ultraviolet radiation [2], contributing to the signaling that controls gene expression [3], iron uptake [4], and photosynthesis [5].

The industry is interested in natural pigments since they are safer and more biodegradable than synthetic pigments for human use [6]. Despite the variety of natural pigments, microbial pigments are preferred due to their easy and quick scaling up and pigment extraction. According to Narsing Rao et al. Numan et al. Celedón and Dáz, and other researchers, the safety of bacterial pigments like melanin, pyoverdine, violacein, carotenoids, prodigiosin, rhodopsins, indigoidine, and pyocyanin prompts their usage in the pharmaceutical, cosmetic, food, and textile industries [6–8]. Additionally, the potential medicinal use of bacterial pigments is strongly associated with their antioxidant, antimicrobial, cytotoxic, and anticancer activities [8].

Pyocyanin is a blue phenazine pigment that is produced by 90–95% of *Pseudomonas aeruginosa* strains [9]. Due to its excellent physical-chemical and biological characteristics, pyocyanin can be used in a variety of biotechnology, engineering, and biological fields. Pyocyanin pigment offers a number of benefits, including being natural and biodegradable, being able to maximize production using inexpensive substrates, and having rapid and easy collection and extraction procedures compared to other chemical synthesis processes [10]. In this review, the production of pyocyanin from *P. aeruginosa* and its extraction process, as well as the application of pyocyanin in many fields, such as aquaculture, agriculture, biosensors, and medicine, are explained in detail.

### *Pseudomonas aeruginosa* as a cell factory for pyocyanin

*P. aeruginosa* is a ubiquitous gram-negative rod that can thrive on basic and low-cost substrate culture media. The formation of the two primary pigments, blue (pyocyanin) and yellow (fluorescein), which lead to the green colorization of the culture plate as well as the sweet aroma of grapes due to the synthesis of 2-aminoacetophenone, is related to the laboratory cultivation of *P. aeruginosa* [11]. The expression of virulence factors in *P. aeruginosa* is enhanced by pigment synthesis, especially pyocyanin. In addition, pyocyanin-producing strains are more virulent and more resistant to many drugs than non-pyocyanin-producing ones [12].

The creation of extracellular polymeric substances, which are mostly made up of polysaccharides, proteins, lipids, and extracellular DNA (eDNA), is necessary for the establishment of both bacterial adhesion and biofilms. The eDNA is a key component of biofilms in *P. aeruginosa* and is crucial for their production and maintenance [13]. Because the pyocyanin-eDNA complex interferes with the cell’s hydrophobicity and fosters the development of robust biofilms, pyocyanin has a significant impact on biofilm formation [14]. Additionally, Das and Manefield speculate that pyocyanin might encourage the release of eDNA in other bacterial species that coexist with *P. aeruginosa* in mixed biofilms [13]. Moreover, pyocyanin is linked to iron uptake by *P. aeruginosa* even in low oxygen conditions [15]. As well, pyocyanin controls the genes elaborated in efflux pumps, which increase the metal’s resistance, especially silver [16].

**Fig. 1:**
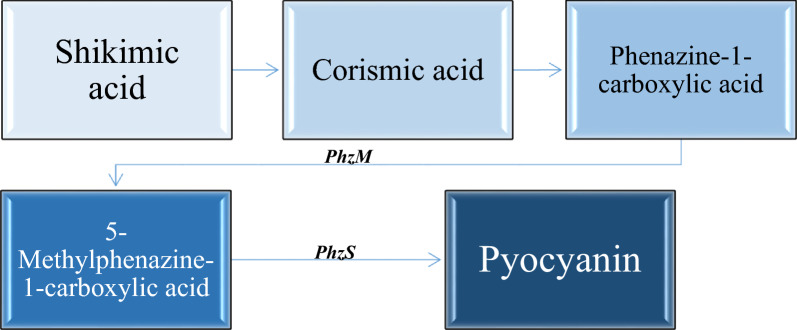
Illustrative scheme showing the pyocyanin synthetic pathway in *P. aeruginosa* starting from shikimic acid

Chorismic acid, which is synthesized from shikimic acid via the *aro* pathway [17], is the pioneer molecule for the synthetic pathway of pyocyanin. Upon the formation of phenazine-1-carboxylic acid from chorismic acid, the synthesis of pyocyanin is regulated by two main genes: phenazine-specific methyl transferase (*phzM*) and flavin-dependent monooxygenase (*phzS*). Phenazine-1-carboxylic acid is converted to 5-methyl phenazine-1-carboxylic acid betaine (MPCBA), by means of a *PhzM*. MPCBA is catalyzed by *PhzS*, involving the hydroxylation of MPCBA to pyocyanin, as shown in Fig. 1 [18].

**Fig. 2:**
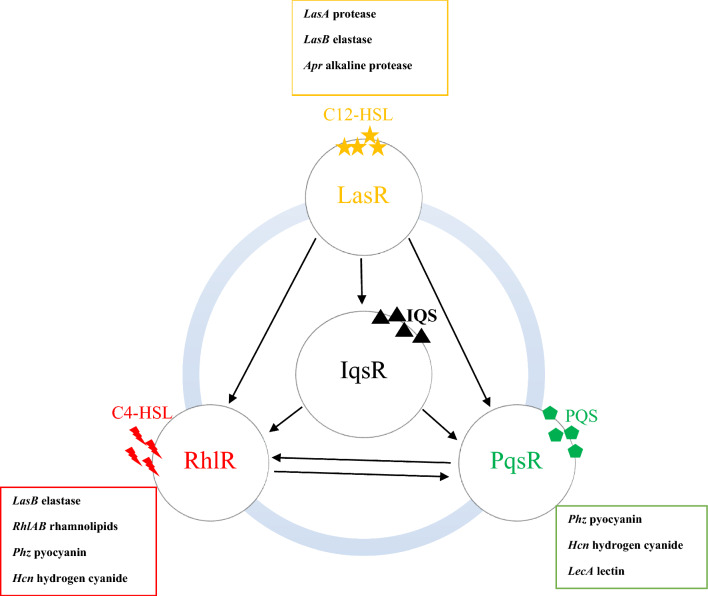
The QS systems in *P. aeruginosa* and their role in pyocyanin production. Pyocyanin is synthesized via multiple gene products encoded by the phenazine synthesis operon, which is regulated by four QS systems in *P. aeruginosa*. The PqsR and RhlR directly control pyocyanin production by activating the phenazine synthesis operon, while the LasR and IqsR indirectly affect pyocyanin production via their activation effects on the PqsR and RhlR (the black arrows)

The quorum sensing (QS) mechanism in *P. aeruginosa* regulates the synthesis of pyocyanin, as shown in Fig. 2 [19]. The QS is a system that relies on data from the bacterial population that is transmitted by tiny, diffusible molecules known as autoinducers, which are separately created by each cell [20]. Acyl-homoserine lactone (AHL) and Pseudomonas quinolone signal (PQS) are the two primary autoinducers of *P. aeruginosa*; both play a remarkable role in pyocyanin expression and regulation [21]. Additionally, the bacterium produces more pyocyanin as a result of an increase in AHL content. The LasR-LasI and RhlR-RhlI systems are the two primary QS systems of *P. aeruginosa*, which activate numerous bacterial expressions, including the creation of alginate, rhamnolipids, and pyocyanin [18]. Zeng and coauthors reported that heat shock proteins (HSPs) play a vital role in pyocyanin synthesis. The role of HSP DnaJ was emphasized by the reduced pyocyanin production in the dnaJ mutant bacteria, which was a result of the decreased transcription of phenazine synthesis operons including *phzA1*, *phzA2*, *phzS*, and *phzM* [22]. Furthermore, pyocyanin synthesis relies on the expression of the cysteine-rich protein (PmtA), which is identified as a metallothionein protein family member, and the expression of *phzM* was decreased in the PmtA mutant during the early stationary phase [23].

### Laboratory production of pyocyanin

**Fig. 3:**
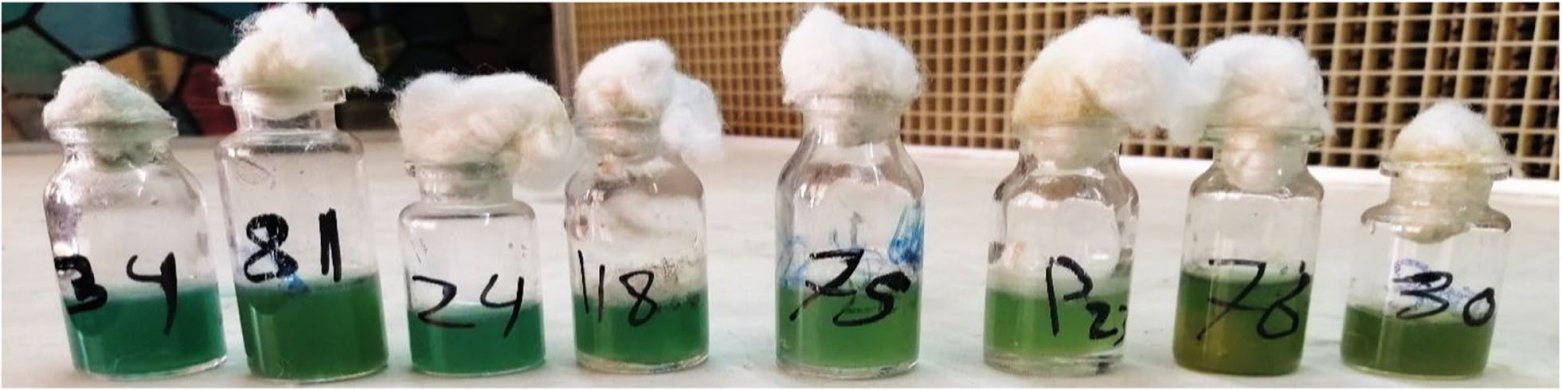
The production of pyocyanin pigment from different *P. aeruginosa* strains at the same incubation conditions in King’s A medium showed that the amount of pyocyanin was different between various strains

**Fig. 4:**
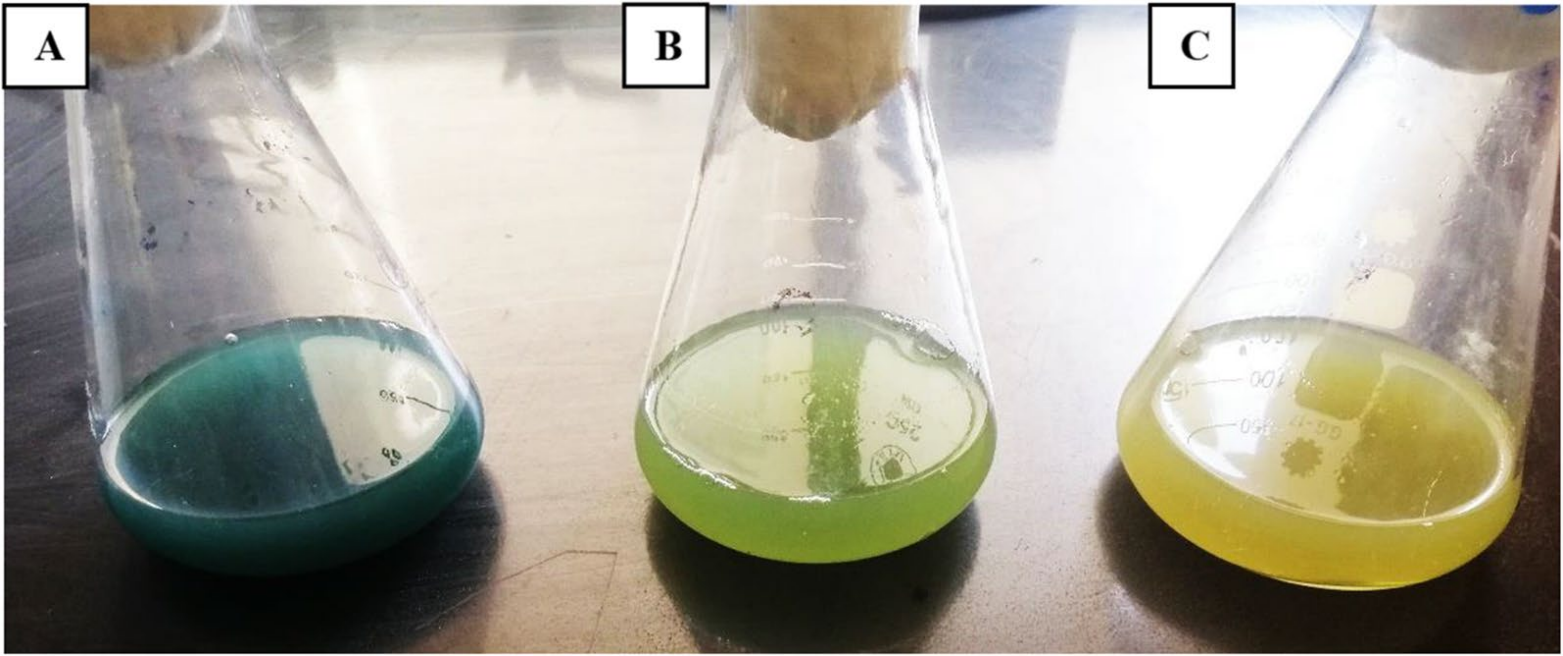
The effect of incubation media composition on the production of pyocyanin from the same *P. aeruginosa* strain **A** peptone water medium **B** King’s A medium **C** nutrient broth medium

King’s A broth was reportedly utilized as a reference culture medium for the detection and synthesis of pyocyanin [18]. The quantity of pyocyanin pigment varies for the same strain and between various stains, as shown in Figs. 3 and 4. The greatest concentration of pyocyanin that may be produced is stable for a single strain of *P. aeruginosa*. The limiting extrinsic factors for regulating the amount of pyocyanin are the level of nutrients and variations in pH, temperature, and aeration [24]. Therefore, numerous studies seek to increase the amount of pyocyanin production in the laboratory by altering the incubation conditions or adding other substances.

The effect of nitrogen source was discussed by Gahlout et al. [25], showing that nitrogen source played a significant role in pyocyanin production, and peptone was found to be the best nitrogen source for pyocyanin production (19.25 µg/ml). Organic nitrogen sources such as urea (13.4 µg/ml) and beef extract (12.2 µg/ml) and inorganic nitrogen sources such as ammonium chloride (12.9 µg/ml) and ammonium sulfate (11.79 µg/ml) showed comparable amounts of pyocyanin production when used alone in the production medium. Similarly, El-Fouly et al. [26] illustrated the production of maximum pyocyanin of 12 mg/ml and 9.3 mg/ml from two different strains of *P. aeruginosa* when peptone was utilized as a nitrogen source in the production medium. Furthermore, the mineral salt medium and peptone water were much better support media for producing blue-green pigment [27].

The synthesis of pyocyanin was affected by the carbon source in the medium [28]. The effect of the carbon source was discussed, and the carbon type had a dramatic effect on pyocyanin production [29]. Sucrose was a much better substrate for pyocyanin production compared to glycerol and glucose. While a study conducted by Abdelaziz et al. [24] showed that using cetrimide as a carbon source was accompanied by enhanced pyocyanin production. In another study, the maximum yield of pyocyanin was obtained with mannitol as a carbon source, followed by glycerol and maltose, while a production medium without any carbon source resulted in a decreased amount of pyocyanin production [25].

The effect of surfactants and organic solvents on the production of pyocyanin was reported by Murat Ozdal [30], who found that Tween 20 and Triton X-100 had an increasing effect on the production of pyocyanin from *P. aeruginosa* OG1. As well, the production of pyocyanin was improved by 125.4 and 275% when chloroform (0.3%, v/v) and toluene (0.2%, v/v) were used in the production medium, respectively. Gahlout et al. [25] studied the effect of inorganic salts on pyocyanin production; the highest amount of pyocyanin pigment was observed when the production medium was supplemented with NaCl. According to Özcan et al. [29], the pyocyanin yield reached its highest value after the addition of 50 mM CaCl_2_ to the culture media.

The impact of metal ions on pyocyanin production was discussed by Gahlout et al. [25], who found that the addition of FeSO_4_ improved pyocyanin pigment production. Methew et al. [31] showed enhancement of pyocyanin yield upon supplementation of the production medium by FeCl_3_ (10 µM). The synthesis of this pigment also appeared to be under the control of iron concentration since the addition of iron to a medium containing low phosphate stimulated the synthesis of pyocyanin [28]. As well, the addition of ZnO nanoparticles to the production medium, in sub-lethal concentrations, served as zinc cation donors and led to increased pyocyanin production [32]. According to Jabłońska et al. pristine multi-walled carbon nanotubes exhibited a stimulative effect on pigment production when applied in high concentrations (500.00 µg/ml) [32]. Recent developments in bioprocessing showed the use of electromagnetic fields (EMFs) to induce the growth of microorganisms and even control the concentration of bioproducts. The 6-h exposure to EMFs resulted in the highest pyocyanin production in comparison to the control [32].

The optimum pH for pigment production ranged from 7.4 to 8.4, but not less than 6.0 or greater than 9.0 [18], while the best incubation period was 72 h, as discussed in previous studies [24, 33]. Pyocyanin production by *P. aeruginosa* began after 10 h of incubation, and it gradually rose to reach its peak yield after 72 h. In order to prevent other pigments from being produced, such as pyomelanin (light brown), pyorubin (red brown), and pyoverdine (green yellow), which could have slowed down pyocyanin production and hampered its extraction, longer incubation time of *P. aeruginosa* was avoided. The agitation conditions affected pyocyanin production; *P. aeruginosa* shaking during incubation increased pigment synthesis [34]. According to Abdelaziz et al., shaking conditions increased pyocyanin production by 31–63.5% compared to static incubation [24]. As well as growth at 37 °C, which was optimal for pyocyanin production [24, 34].

Herein, we mentioned some studies that fabricated a different system for batch fermentation of pyocyanin and the maximization of its yield. A modified semisynthetic medium that contains tryptone and yeast extract as a nitrogen source, glycerol, and lactose as a carbon source, along with salts Na_2_HPO_4_, MgSO_4_, KCl, and K_2_HPO_4_, was utilized with agitation conditions at 37 °C and a pH equal to 8, and the produced pyocyanin was approximately 6 µg/ml [35]. The maximum production of pyocyanin by *P. aeruginosa* OG1 (33 mg/ml) was achieved when 0.2% toluene was added to nutrient broth containing 1% glycerol under shaking incubation at 30 °C for 72 h, and the initial medium pH was 7.2 [30]. The submerged fermentation by *P. aeruginosa* NEJ01R using lime-cooked maize wastewater (nejayote) in Luria-Bertani medium at 29.6 °C, 223.7 rpm, and a pH equal to 6.92 produced a pyocyanin yield reaching a value of 3.25 µg/ml [36]. During the optimization of pyocyanin production of *P. aeruginosa* DN9 [25], maximum pyocyanin production (92.12 µg/ml) was obtained with carbon source mannitol, nitrogen source peptone, inorganic salt NaCl, and metal ion FeSO_4_ at 30 °C under shaking conditions at 120 rpm for 48 h. The pH-adjusted (pH= 8) peptone water medium containing 3% (w/v) cetrimide was used, while *P. aeruginosa* strain P 32 was incubated using an incubator shaker for 3 days at 37 °C, and the yield of pyocyanin was 53 µg/ml [24].

In conclusion, the regulation of the extrinsic factors that control pyocyanin production, such as nutrients, pH, temperature, shaking incubation, and aeration, using the abovementioned batch fermentation systems greatly improved pyocyanin yield, especially when highly pyocyanin-producing *P. aeruginosa* strains were utilized in the fermentation process. In the next studies, it will be interesting to explore the effect of the combination of various carbon sources, such as mannitol and cetrimide, on pyocyanin production. Additionally, studying the impact of using two or more pyocyanin-producing *P. aeruginosa* strains in the same fermentation system instead of one strain may be promising in the future studies.

### Extraction of pure pyocyanin

**Fig. 5:**
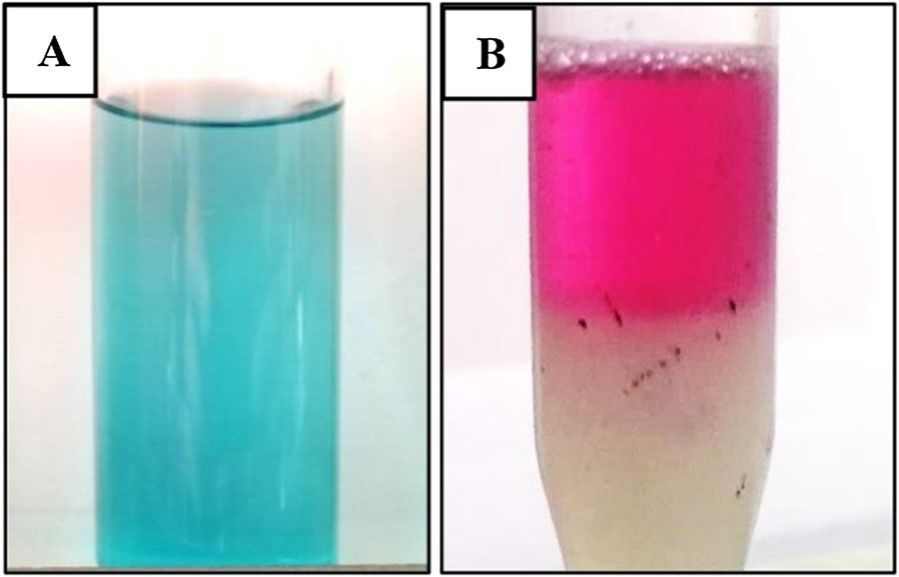
The dualism of solubility of pyocyanin in organic (**A**) and aqueous (**B**) solvents with its characteristic pH-dependent color change

The solubility of pyocyanin fluctuates with changes in the pH of the solution, as shown in Fig. 5. At normal and alkaline pH, pyocyanin is soluble in organic solvents, such as chloroform, and has its distinctive blue color. At a lower pH, it is easily soluble in the aqueous phase and changes color to a reddish pink. By adjusting the pH and using water/chloroform extraction, this duality in solubility makes the purification of pyocyanin promising and extremely successful [24, 33, 35, 37].

**Fig. 6:**
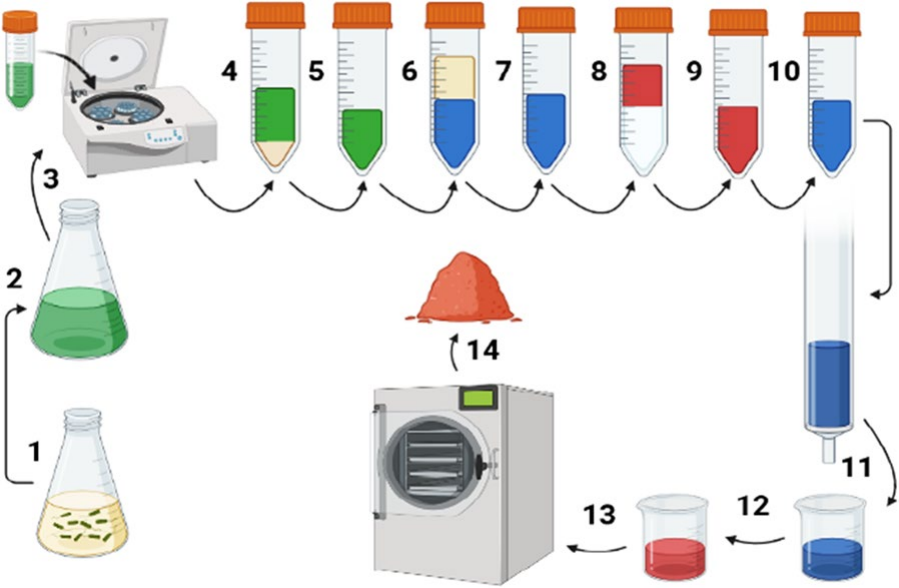
Schematic diagram showed the steps of pyocyanin production and extraction from *P. aeruginosa* until obtaining a stable red pyocyanin powder

Herein, the extraction steps of pyocyanin were illustrated according to the previous Reference: Kindly check whether the inserted [DOI] for references [28, 38, 45, 78, 92, 96, 100] are appropriate. studies to facilitate and summarize how to obtain a pure and stable pyocyanin [24, 33, 35, 37], as shown in Fig. 6. After the incubation of *P. aeruginosa* in the selected medium for pyocyanin production and incubation at optimal conditions (Step 1), the color of the medium became dark green due to the generation of pyocyanin pigment (Step 2). For the removal of bacterial cells, the culture medium was subjected to centrifugation at 12,000*g* for 20 min at 4 °C (Step 3). The resultant supernatant (Step 4) was filtered through a 0.2 µ syringe filter to obtain the clear and green solution of pyocyanin (Step 5). The green pyocyanin solution was mixed with an equal amount of chloroform and vortexed for 20 s for color change (Step 6). The pyocyanin-free medium on the top layer was discarded, and the chloroform layer was transferred to another new falcon tube (Step 7). An equal volume of 0.2 N HCl was added and subjected to centrifugation at 10,000*g* for five min (Step 8). During step 8, pyocyanin moved to the 0.2 N HCl layer, the top layer, and its color changed to red. The red pyocyanin layer was transferred to a new falcon tube for further purification (Step 9). To increase pyocyanin purity, 0.2 N NaOH was added drop-by-drop, leading to a change in the pH of the solvent and subsequently changing the pyocyanin color to blue (Step 10). Then, the pyocyanin purification was done using silica gel column chromatography (Step 11). During step 11, the pyocyanin was transferred again to chloroform and loaded into the column (30 cm length and 2 cm diameter). The column was packed with silica, having a mesh size of 200–500, and equilibrated using a 1% chloroform-methanol solvent system in a ratio of 1:1. When pyocyanin was eluted with 15% methanol in chloroform, crude pigment fractions on the silica gel column appeared in yellow-green, light blue, and dark blue bands. The blue fractions were collected, protected from light, and analyzed by a UV-Vis spectrophotometer in comparison to standard pyocyanin. The collected blue pigment was transferred again to 0.2 N HCl (Step 12) and subjected to freeze-drying with subsequent exposure to a vacuum for 24 h after freezing at − 80 °C for 12 h using 1% sorbitol as a cryoprotectant (Step 13). The obtained red pyocyanin powder (Step 14) can be stored at − 18 °C for further application.

### Pyocyanin stability

**Fig. 7:**
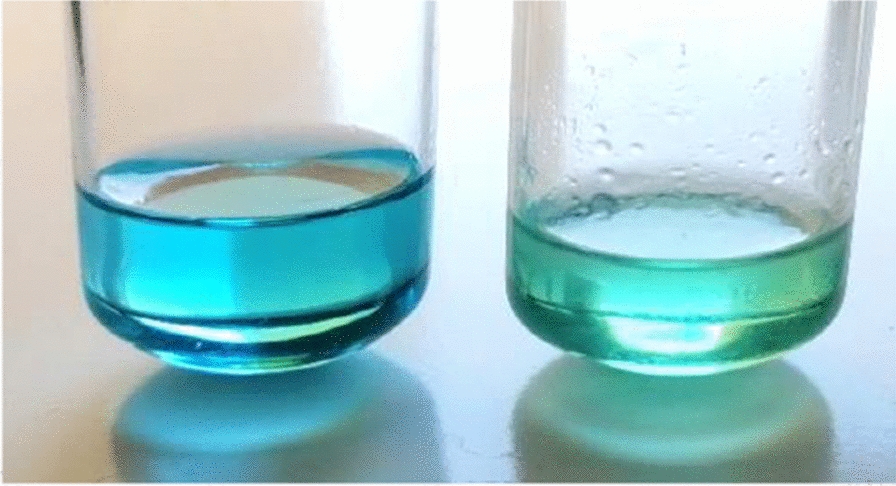
Loss of pyocyanin’s characteristic blue color upon degradation

The degradation of pyocyanin is easily noticed by the disappearance of its characteristic blue color [24], as shown in Fig. 7. Abou and Feghali observed many stability problems during the preparation of pyocyanin crystals [33]. After heating, by putting the solution in a water bath and subsequently chloroform evaporation, the obtained pyocyanin crystals could not be separated from the impurities that crystalized with it. However, this attempt was also unsuccessful because pyocyanin was degraded in the process. On the other hand, using the rotary evaporator led to the formation of water-insoluble crystals, although pyocyanin is a water-soluble pigment, due to the crystallization of chloroform with the pyocyanin and preventing its contact with water.

Additionally, the kind of solvent used to store pyocyanin had an impact on its stability over time. The stability of pyocyanin was found to be preserved for a year when it was stored in its lyophilized form at − 18 °C, but it degraded after 60, 29, and 15 days in sterile distilled water (SDW), 0.2 N HCl, and chloroform, respectively, when it was stored in its dissolved form at − 18 °C [24]. Pyocyanin only lasted for two days in dry chloroform before its deterioration, compared to 12 days in water, according to Abou and Feghali’s findings. Pyocyanin’s stability in 80% methanol was also comparable to that of pyocyanin in water at the same solute content [33]. According to Abdelaziz et al., the degradation rate was quicker in chloroform and 0.2 N HCl in comparision with SDW [24]. The stability of pyocyanin was dependent on the surrounding temperature; it was evident that pyocyanin was degraded progressively with increasing temperatures [24]. In fact, pyocyanin was stable for 12 days at a temperature of 25 °C, whereas it was stable for only 1 day at a temperature of 70 °C (in a water bath), 8 days at 40 °C, and 2 days at 50 °C [33].

### Pyocyanin and oxidative stress

**Fig. 8:**
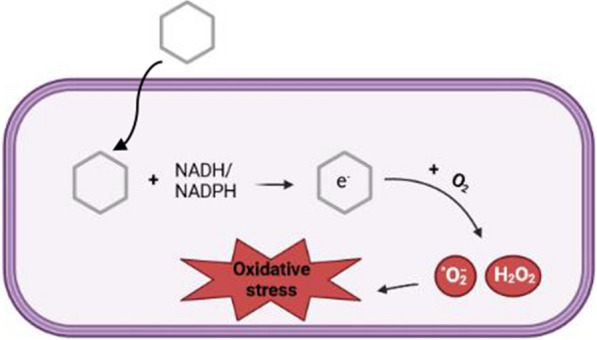
The production of ROS by pyocyanin and subsequently oxidative stress formation. A six-membered ring represents pyocyanin

Reactive oxygen species (ROS) levels that are significantly higher than antioxidant levels are considered to be an indicator of oxidative stress [38]. Through the passage of electrons and the formation of ROS after interaction with molecular oxygen, pyocyanin exerts its effects by inducing oxidative stress in sensitive prokaryotes and eukaryotes [39]; Pyocyanin merely enters cell membranes, and takes electrons from NADPH or NADH, which leads to ROS, particularly superoxide (O_2_^−^) and hydrogen peroxide (H_2_O_2_), production when it transfers those electrons to oxygen in an aerobic environment [40, 41], as shown in Fig. 8. It has been shown that *P. aeruginosa* strains that overproduce pyocyanin produce higher levels of oxidative stress, which leads to cell lysis and a significant increase in eDNA, supporting the idea that oxidative stress plays a crucial role in pyocyanin’s ability to induce cytotoxicity [42]. Pyocyanin exposure causes the creation of intracellular ROS in host cells, which leads to DNA damage specifically [43], as well as oxidative damage to cell cycle components, NAD(P)H depletion, and enzymatic inhibition [42]. In eukaryotes, increased ROS speeds up aging and apoptotic processes [44]. In prokaryotes, Iiyama et al. reported that pyocyanin’s ability to kill microorganisms is mediated via the production of ROS, which results in the inhibition of the ion’s interaction with the membrane, and respiration [45].

### Applications of pyocyanin

**Fig. 9:**
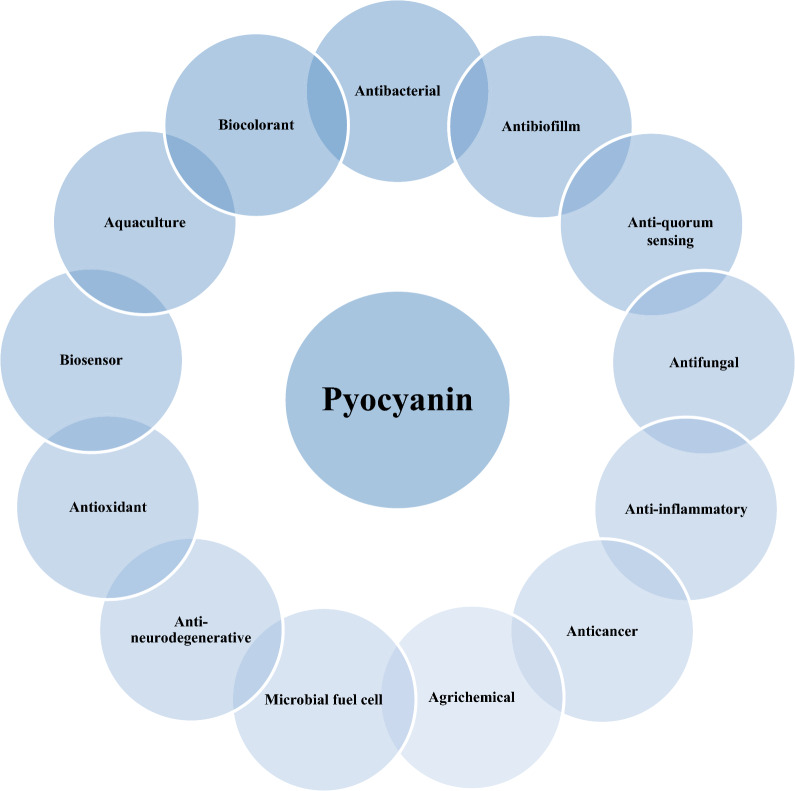
The compilation of the applications of pyocyanin in many fields such as medicine, agriculture, aquaculture, and biosensors

Pigments that are produced as secondary metabolites to protect microorganisms from harmful effects also have several biological activities [6, 8, 9]. In this review, we have discussed the applications of pyocyanin (Fig. 9) and exposed that it holds the promise to be applied as a potential agent in different areas, including aquaculture, agriculture, biosensors, and medicine. However, further studies are indeed necessary to reveal its new functions.

### Pyocyanin as an antibacterial agent

Many studies have discussed the antibacterial activity of pyocyanin against both gram-positive and gram-negative bacteria. El-Fouly et al. [26] observed the growth inhibition activity of pyocyanin against *Staphylococcus aureus*, *Escherichia coli*, *Klebsiella* species, *Salmonella typhi*, and *Shigella* species. According to Abdul-Hussein and Atia [27], the most sensitive bacteria to pyocyanin were *E. coli*, followed by *Bacillus* and *Staphylococcus* at the same level. The results of the study conducted by Darwesh et al. [46] indicated that pyocyanin had high killing activity against all tested bacteria except *Shigella species*. Aziz and coauthors found that pyocyanin inhibited the growth of *S. aureus*, *K. pneumonia*, *Enterococcus faecalis*, *Burkholderia cepacia*, and *E. coli* [47]. According to Hamad et al. [48], pyocyanin exerted antibacterial activity against *Bacillus* cereus, *S. aureus*, *Staphylococcus sciuri*, *E. coli*, *S. typhi*, *Salmonella enterica*, *K. pneumonia*, and *Lactococcus lactis*. The antibacterial activity of pyocyanin against foodborne pathogenic bacteria such as *Bacillus spizizenii*, *S. aureus*, *Enterobacter aerogenes*, *S. enterica*, and *E. coli* was reported by Saleem and coauthors [37]. Lyophilized pyocyanin showed dose-dependent antibacterial activity against methicillin-resistant *S. aureus* (MRSA) and accelerated the wound healing process in the rat wound infection model [49]. Moreover, the gene expression analysis revealed that pyocyanin remarkably inhibited the efflux pump gene, the *smr* gene, in *Serratia marcescens*. As is known, efflux pumps remove drugs, making infections more difficult to treat. Therefore, pyocyanin can be used against multidrug-resistant gram-negative bacteria by inhibiting efflux pumps [50]. Additionally, the synergistic activity of pyocyanin with some antibiotics was reported; *S. aureus* became sensitive to novobiocin and nalidixic acid when mixed with 1 mg/ml pyocyanin. While *E. coli* became sensitive to nalidixic acid and ciprofloxacin when mixed with 100 mg/ml pyocyanin [51]. Dhairh and Al-Azawi showed that the pyocyanin-meropenem combination was more effective than meropenem alone for *Proteus mirabilis* and *S. marcescens* [52].

### Pyocyanin as an antifungal agent

The bacterial pigment pyocyanin reduced the growth of many fungi; *Aspergillus niger* and *Aspergillus fumigatus* were the most affected, followed by the yeast *Cryptococcus neoformans*, while *Candida tropicalis* and *Candida albicans* were equally affected. On the other hand, *Candida krusei* was least affected by pyocyanin [27]. Darwesh et al. [46] reported that the growth of *C. tropicalis*, *Candida albicans* ATCC-10231, and *Fusarium oxysporum* was inhibited by pyocyanin with an inhibition zone of 34, 35, and 15 mm, respectively. DeBritto and coauthors noticed that the growth of *Magnaporthe grisea* fungus was completely inhibited at 200 ppm of pyocyanin [53]. The broad-spectrum antifungal activity of pyocyanin was discussed by Hamad et al. who found that pyocyanin was effective against *Aspergillus flavus*, *Aspergillus carbonarius*, *Aspergillus stynii*, *Fusarium verticillioides*, *Fusarium proliferatum*, and *Penicillium verrucosum* at a concentration of 0.1 mg/ml [48]. Moreover, Saleem et al. showed that pyocyanin at 50 µg/ml was effective against *F. oxysporum*, *A. niger*, and *A. fumigatus* [37].

### Pyocyanin as an antibiofilm agent

Biofilms are complex, three-dimensional communities of microorganisms that are adhered to surfaces and covered in exopolymeric material. Several bacterial pathogens, such as *P. aeruginosa* [54], *S. aureus* [55], and *E. coli* [56], are believed to produce biofilms as a crucial component of their pathogenicity during the pathogenesis of their diseases. As well, it has been determined that biofilms account for 80% of all microbial diseases in humans [57]. Bacteria in biofilms are inherently more resilient to antimicrobial treatment as compared to planktonic cells of the same bacterial strain. According to studies, biofilm-forming bacteria are typically thousands of times more resistant to antimicrobial treatment than their planktonic relatives [57]. For instance, naturally drug-susceptible bacterial strains commonly exhibit substantial antibiotic resistance when they are in the biofilm mode of life [58]. Theoretically, various pathways for bacterial antimicrobial resistance are involved in biofilm antibiotic tolerance. The extracellular polymeric substance of numerous biofilm-forming species has demonstrated an innate capacity to prevent antibiotic penetration [59]. It is well known that the complex internal structure of a biofilm produces microenvironments devoid of oxygen and nutrients [60], and a lack of oxygen and nutrients is known to limit bacterial growth and increase antibiotic resistance in many species [61].

The notion of using phenazines to treat biofilm came from the ability of *P. aeruginosa* biofilm in the lungs of cystic fibrosis patients to outcompete *S. aureus* biofilm with the aid of pyocyanin [62]. Pyocyanin extracted from *P. aeruginosa* BTRY1 exhibited a remarkable reduction in foodborne pathogens’ biofilm formation belonging to the genera *Bacillus*, *Staphylococcus*, *Brevibacterium*, and *Micrococcus* [63]. Using a crystal violet assay, Saleem et al. reported that pyocyanin inhibited biofilm formation by *B. cereus*, *S. aureus*, and *K. pneumonia*. As well, disruption of pre-formed biofilm against *B. cereus*, *S. aureus*, and *K. pneumonia* was observed [37]. Kamer and coauthors reported that pyocyanin inhibited the growth of MRSA isolates in their biofilm mode, and the mechanism by which pyocyanin eradicated biofilm was associated with a reduction of the exopolysaccharide matrix and bacterial viability within the established biofilm after pyocyanin treatment [49].

### Pyocyanin as an anti-quorum sensing agent

Cell-cell communication, or QS, is a widespread phenomenon in bacteria that is used to coordinate gene expression among local populations. Its use by bacterial pathogens to regulate genes that promote invasion, defense, and spread has been particularly well documented [64]. Driven by the concerning global spread of antibiotic resistance and the ongoing difficulties of finding a working vaccine, there has been an increase in the recent interest in the development of alternative treatment strategies for bacterial infections. Anti-virulence strategies targeting QS (QS blockers) represent an efficient pathway for combating bacterial infection with a smaller risk of the development of resistance [65].

The primary controller of QS, which in turn controls a number of *S. aureus* virulence factors, is the Agr system [66]. The AgrA protein is a component of the Agr system that, upon stimulation, binds to P2 or P3 promoters, upregulating a number of virulence factors with significant effects on pathogenicity [67]. Researchers’ primary focus was therefore on decreasing *S. aureus* pathogenicity rather than its survival [68–70]. Competing for *S. aureus* pathogenicity by blocking Agr QS occurred by interfering with AIP binding to AgrC from the extracellular space, such as with AIP analogs and several natural compounds, including fengycins. The other way is to penetrate the cytoplasm and inhibit the response regulator AgrA protein, such as with savirin or apicidin [65]. Pyocyanin demonstrated its anti-Agr QS action, according to Kamer et al. [49], by reducing Agr-dependent phenotypes such as spreading motility, hemolytic activity, proteolytic activity, and the downregulation of the *agrA* gene transcription. The *in-silico* investigation also showed that pyocyanin bound to the AgrA protein at a crucial position, which was necessary for appropriate folding, DNA binding, and the exertion of its QS regulatory action [71]. Pyocyanin’s strong anti-QS activity was highlighted by the number of amino acid residues involved in its binding to AgrA protein, which was greater than the number of amino acid residues involved in previously published Agr-mediated QS inhibitors like salicylic acid, Azan-7, and staquorsin [67, 72, 73].

### Pyocyanin as an anticancer agent

The ability of pyocyanin to influence many cancer types has been discussed in numerous studies. Hassani et al. [74] reported that pyocyanin, which was produced by mutant strain S300-8, revealed a powerful efficiency against the growth of a rhabdomyosarcoma cancer cell line, whereas the cytotoxicity was improved by increasing the concentration of pigment with a period of exposure time. According to Zhao et al. pyocyanin could affect hepatocellular carcinoma HepG2 cells, which was mediated by acute ROS production and subsequent oxidative stress, DNA damage, and activation of caspase-3, as well as accelerating cell senescence and apoptosis [41]. Additionally, it has been shown that cancer cells can be prevented from growing by using the biological features of the pyocyanin pigment, such as cell permeability induction, DNA intercalation, and topoisomerase inhibition [75]. In the study conducted by Moayedi et al. pyocyanin could also induce dose-dependent apoptosis and necrosis in human pancreatic cancer Panc-1 cells after 24 h [76]. Sengupta and Bhowal [77] determined that the pyocyanin pigment showed cytotoxic activity against human osteosarcoma cells MG-63 on day 1 after exposure to pyocyanin, which was recorded to be 85.57% and gradually decreased to 11.01% at the end of day 5. The study established by Abdelaziz et al. reported that pyocyanin was cytotoxic against human breast adenocarcinoma MCF-7, with an IC_50_ equal to 15 µg/ml, by inducing apoptosis and necrosis, while the cells of MCF-7, after pyocyanin treatment, became shrunken in size, round, and disconnected from the monolayer surface of the wells [24]. The anti-proliferative action of pyocyanin pigment on human melanoma cells (SK-MEL-30) and human colon cancer cells (HT-29) was reported by Koyun et al., and it increased with increasing pyocyanin concentration. In addition, the IC_50_ values of the pyocyanin pigment for SKMEL-30 and HT-29 were 72 µM, and 179 µM, respectively [38].

### Pyocyanin as an antioxidant agent

An antioxidant is a molecule that is able to slow or prevent the oxidation of other molecules, which leads to the formation of free radicals [47]. Microbial pigments have unlimited potential for application as an antioxidant agent due to their safety, low cost of production, and free radical scavenging capacity [78, 79]. Aziz et al. reported that pyocyanin pigment is highly active as an antioxidant by converting the dye color of 2, 2-diphenyl-1-picrylhydrazyl (DPPH) to a dark yellow color, and the maximum antioxidant activity was accomplished at a concentration of 1.125 mg/ml [47]. On the other hand, Saleem et al. found that pyocyanin at a concentration of 50 µg/ml showed an antioxidant potential with 58% inhibition of DPPH radicals and 52.5% free radical scavenging of 2,20-azinobis-3-ethylbenzothiazoline-6-sulfonic acid [37]. Additionally, Sengupta and Bhowal reported the DPPH radical scavenging activity of pyocyanin with an IC_50_ value equal to 4.75 µg/ml [77].

### Biocolorant agent

Dyes are utilized in many industrial products, such as cosmetics, food, pharmaceuticals, textiles, paper printing, and plastic industries [80]. Water contamination by dyes is dangerous for aquatic organisms and humans, as dyes are carcinogenic and mutagenic to humans [81]. Additionally, the perfect remediation technology to remove such pollutants is unavailable [82]. Therefore, to lessen the harmful effect of synthetic dyes, natural pigments can be applied as a substitute, as natural pigments are considered safe, not carcinogenic, and biodegradable in nature [25]. DeBritto et al. [53] discussed the dye nature of pyocyanin; the treatment of cotton cloth with pyocyanin pigment resulted in a permanent color change of the cotton from white to pink. As well, Gahlout and coauthors reported that the methanol solution of pyocyanin was an efficient textile colorant [25].

### Agrichemical agent

The imbalance in agricultural soil has been brought on by the widespread use of chemicals in agriculture, such as pesticides and fertilizers. Numerous other approaches have been proposed for use. In recent research, the utilization of microbial products rather than microbial cells to improve plant growth has been the main focus [83]. It was found that pyocyanin was necessary for the nematode *Caenorhabditis elegans* and the fruit fly *Drosophila melanogaster* to be killed, as well as for the prevention of disease signs in plants [84]. Additionally, pyocyanin from *Pseudomonas* species isolated from rhizosphere soil was employed as a biocontrol agent against *Fusarium*, the cause of chickpea wilt and Pythium damping of beans [85]. It was discovered that the pyocyanin produced by the *P. aeruginosa* PUPa3 strain exhibited biocontrol activity against a variety of phytopathogenic fungi that affect the growth of rice, groundnut, tobacco, chili, mango, sugarcane, tea, cotton, and banana crops [86]. De Vleesschauwer et al. [87] reported using pyocyanin from the *P. aeruginosa* 7NSK2 strain as a biocontrol agent against the *Magnaporthe grisea* leaf blast and *Rhizoctonia solani* sheath blight in the monocot model rice plant. According to Khamdan and Suprapta [88], *P. aeruginosa* enhanced yield, chlorophyll content, and peroxidase activity and acted as a very effective biopesticide against soybean stunt virus. Pyocyanin has been shown to be antagonistic to the plant pathogenic fungus *Macrophomina phaseolina*, which causes stem rot, seedling blight, and root rot, according to De Britto et al. [53]. When pyocyanin was applied to the soil, some nutrients and minerals, like magnesium, chlorine, and iron, significantly increased, and these amounts gradually increased as the pyocyanin concentration increased [83].

### Pyocyanin-based aquaculture

One of the most widely used viable aquaculture production techniques is recirculating aquaculture systems (RAS). Biological filters, which are a crucial component of the RAS, are vulnerable to antibiotics or other antibacterial treatments as nitrifying bacteria are used in them. Therefore, newer medications that don’t interfere with their action are needed for the successful control of illnesses in RAS [89]. Pyocyanin was found to considerably reduce the population of *Vibrio* spp. in RAS at a concentration of 5 mg/L without appreciably influencing the activity of the nitrifying bacteria utilized in RAS. In addition, the raised shrimp (*Penaeus monodon*) demonstrated 100% survival after the application of pyocyanin [90].

In the African catfish hatchery in Nigeria, saprolegniasis is a serious pathogenic infection that drastically lowers the hatching rates of fish eggs that have been incubated. Microscopic examination of the unhatched eggs after pyocyanin treatment showed that a pathogenic fungus called *Saprolegnia* sp. and a protozoan ciliate known as *Colpidium* sp. that were present in the control were absent. In addition, after cultivating unhatched eggs from the control, Sabourand dextrose agar demonstrated the growth of *Saprolegnia* sp. that was not found in case of pyocyanin treatment [91].

### The anti-neurodegenerative activity of pyocyanin

Progressive neuronal loss, motor and cognitive impairment, and aberrant protein aggregation are all symptoms of Alzheimer’s disease (AD) and other neurodegenerative disorders [92]. Abnormal acetylcholinesterase (AChE) expression is intimately associated with a number of neurodegenerative diseases, as the elevated AChE enzyme activity leads to a decrease in acetylcholine (Ach) levels and subsequently the fast termination of nerve impulses and neural loss [93]. As a result, the major method used to treat AD symptoms is to enhance cerebral Ach levels by inhibiting AChE activity, which will improve cholinergic signal conduction [94]. Garcia-Ratés et al. reported the AChE inhibitory activity of pyocyanin, which increased with increasing pyocyanin concentration and reached the maximum AChE inhibitory activity at 100 µM [95]. Pyocyanin pigment is believed to have an inhibitory effect on AChE enzyme activity due to its redox-active chemical characteristics and its small size, which allows it to penetrate through cell membranes [38].

### Biosensor

Electrochemical detection techniques of *P. aeruginosa* are possible due to the electroactivity of redox-active pyocyanin pigment, which allows quick detection of *P. aeruginosa*. Voltammetric techniques such as cyclic voltammetry, differential pulse voltammetry, and square wave voltammetry were discussed by McLean et al. for the rapid detection of *P. aeruginosa* [96]. According to Manisha and coauthors, *P. aeruginosa* was electrochemically characterized depending on pyocyanin using disposable paper electrodes, which have substantial advantages as biosensors such as easy batch manufacturing, low cost, and simplicity [97]. According to Alatraktchi, *P. aeruginosa* in water sources was electrochemically detected in only 14 s by its unique biomarker pyocyanin, and the electrochemical response was linearly proportional to the pyocyanin concentration with R^2^ values between 0.9918 and 0.9991 [98]. McLean et al. utilized in-house 3D-printed carbon nanotube electrodes for the rapid detection of *P. aeruginosa* and could detect very low concentrations of pyocyanin (1µM) due to the superior conductivity of multi-walled carbon nanotubes [99]. By using the thermal sensor, Frigoli and coauthors indirectly detected *P. aeruginosa* infection; thermal resistance studies were carried out at clinically relevant concentrations of pyocyanin (1.4–9.8 µM) with a limit of detection of 0.347 ± 0.027 µM, utilizing molecularly imprinted polymer particles attached to planar aluminium chips using adhesive film [100].

### Microbial fuel cells (MFCs)

Devices called microbial fuel cells (MFCs) are designed to use the energy produced by bacterial metabolism; these tools link the energy released when bacteria metabolize their substrates [101]. Normally, the resulting electrons of bacterial metabolism are transferred to a terminal electron acceptor (oxygen in aerobic respiration and organic compounds in anaerobic respiration) to conserve the released energy in the readily available energy storage molecule adenosine triphosphate (ATP) [102]. However, in the case of MFC, highly energized electrons must first be able to pass the cytoplasmic membrane in order to capture their bioelectricity [103]. In most bacteria, this movement across the cytoplasmic membrane is not a normal occurrence, but in electrochemically active bacteria (EAB), this movement across the membrane can be easily accessed, making it possible to collect the electrons inside an MFC [104]. *P. aeruginosa* is an EAB with significant promise since it produces pyocyanin [105], and the increased energy output has been linked to increased pyocyanin synthesis [106]. Since pyocyanin is the primary endogenous electron transfer shuttle, it facilitates the extracellular flow of electrons from bacterial cells to anodes [35]. Moreover, R. Kumar and coauthors reported that pyocyanin increased the electron transfer from *P. aeruginosa* as well as other species used in MFC [107].

### Pyocyanin as an anti-inflammatory agent

A complicated cascade of intracellular signal transduction and transcription processes stimulates macrophages during inflammation [108], and inflammatory mediators like TNF-α and IL-1b are released by activated macrophages [109]. Allen and coauthors reported that the production of IL-6, IL-1b, keratinocyte-derived chemokines, and macrophage inflammatory protein (MIP)-2 decreased in the lungs of C57BL/6 mice infected by *P. aeruginosa* by pyocyanin [110]. Furthermore, Fujihara et al. found that pyocyanin reduced the levels of TNF-α and IL-1b [111]. de Sales-Neto et al. discussed that pyocyanin exerted its anti-inflammatory activity in LPS-activated macrophages by reducing the generation of nitric oxide, IL-1b, and TNF-α without causing macrophage mortality [112].

## Future perspectives

Pyocyanin shows promising antibacterial and antifungal activities against several microbes and also has antioxidant and anticancer activities against cancer cell lines. These findings can be used as the basis for using pyocyanin as a food preservative agent in food industries and in the preparation of topical and, to some extent, systemic antimicrobial agents for treating many microbial diseases. Furthermore, pyocyanin may be used as an adjuvant agent in the treatment protocol of cancer therapy besides chemotherapeutic agents to improve the efficacy of these agents and decrease the probability of resistance. Future bioprospecting is required to uncover new therapeutic traits of pyocyanin pigment, aiming for environmentally-friendly applications in many other fields.

## Conclusion

*Pseudomonas aeruginosa* is the only natural source for the production of pyocyanin pigment. The production of pyocyanin pigment varies between *P. aeruginosa* strains and is affected by several extrinsic factors, such as nutrients, pH, agitation, aeration, and duration of incubation. It is preferred to utilize a highly pyocyanin-producing *P. aeruginosa* strain and modify the extrinsic factors to obtain a great pyocyanin yield. The dual solubility of pyocyanin facilitates its extraction and purification using chloroform, HCl, and NaOH solvents. The physical-chemical properties of pyocyanin enable it to possess many biological activities, such as antibacterial, antifungal, antibiofilm, anti-quorum sensing, anticancer, anti-inflammatory, antioxidant, and anti-neurodegenerative activities. Moreover, pyocyanin can be used in the areas of agriculture and aquaculture, as well as a stable biocolorant agent, a biosensor for the detection of *P. aeruginosa* infection, and an electron shuttle in MFCs to improve electron transfer. Further new studies about pyocyanin are important in the future to investigate its unknown applications.

## Data Availability

All data generated or analyzed during this study are included in this published article.
